# The Latest Fashion on the New Plan

**Published:** 1847-10

**Authors:** 


R. R. KELLOGG,

(FROM NEW YORK,)

SURGEON DENTIST,

☞Inserts Teeth in the latest Fashion on the new plan. He ascertains the number of decayed Teeth by pulsation, and performs a Surgical Operation which will in all cases cure the Tooth Ache. He also plugs and cleans in a very superior manner, and radically removes the cause by which they are destroyed. Satisfaction given or no pay required. Prices to suit the the times. Call and see.

☞TOOTH POWDER always on hand of a very superior quality.

The above card was placed in our hands some two years since, when on our journey East. We had intended an initiation into some of the mysteries of the science as practiced and understood by our brethren of New York, while in that city. But unfortunately for the introduction of *improved* Dentistry in the West, a grave and very important question in relation to the use of Amalgam fillings for the teeth so completely absorbed the attention of the Profession while there, that the other was entirely neglected We are sorry to say that, owing to the premature decease of the *worthy Dr.* entrusted with *"the latest fashion on the new plan,"* from head quarters at New York, they have failed reaching the Queen City. Indeed we are told the practice has not been introduced farther west than the Allegheny—

And there on "Pisgah's top" the Dentist stood,
And viewed the promised land.

But like one of old, failed to enter—and disseminate his useful knowledge, so we are utterly at a loss to know the "latest fashion on the new plan."

The manner of "ascertaining the number of decayed teeth by pulsation" or the particular kind of a "surgical operation which will in all cases cure the tooth-ache," (unless indeed it be the old one of extraction.) It may, however, be an operation on the Toe, the Ear, or the Brain. We have heard of people being sometimes "bored for the simples."

We hope soon to get the above knowledge from the East, also the manner of "radically removing the cause by which teeth are destroyed." We shall then feel that we have labored not in vain.—Cin. Ed.

